# A Novel *TTC19* Mutation in a Patient With Neurological, Psychological, and Gastrointestinal Impairment

**DOI:** 10.3389/fneur.2019.00944

**Published:** 2019-09-04

**Authors:** Parham Habibzadeh, Soroor Inaloo, Mohammad Silawi, Hassan Dastsooz, Mohammad Ali Farazi Fard, Forough Sadeghipour, Zahra Faghihi, Mohaddeseh Rezaeian, Majid Yavarian, Johann Böhm, Mohammad Ali Faghihi

**Affiliations:** ^1^Persian BayanGene Research and Training Center, Shiraz University of Medical Sciences, Shiraz, Iran; ^2^Student Research Committee, Shiraz University of Medical Sciences, Shiraz, Iran; ^3^Neonatal Research Center, Shiraz University of Medical Sciences, Shiraz, Iran; ^4^Italian Institute for Genomic Medicine (IIGM), University of Turin, Turin, Italy; ^5^Institut de Génétique et de Biologie Moléculaire et Cellulaire (IGBMC), Inserm, CNRS, Université de Strasbourg, Illkirch, France; ^6^Center for Therapeutic Innovation, Department of Psychiatry and Behavioral Sciences, University of Miami Miller School of Medicine, Miami, FL, United States

**Keywords:** mitochondrial diseases, mitochondrial encephalomyopathies, *TTC19*, mitochondrial complex III deficiency, neurodegenerative diseases

## Abstract

Mitochondrial complex III deficiency nuclear type 2 is an autosomal-recessive disorder caused by mutations in *TTC19* gene. *TTC19* is involved in the preservation of mitochondrial complex III, which is responsible for transfer of electrons from reduced coenzyme Q to cytochrome C and thus, contributes to the formation of electrochemical potential and subsequent ATP generation. Mutations in *TTC19* have been found to be associated with a wide range of neurological and psychological manifestations. Herein, we report on a 15-year-old boy born from first-degree cousin parents, who initially presented with psychiatric symptoms. He subsequently developed progressive ataxia, spastic paraparesis with involvement of caudate bodies and lentiform nuclei with cerebellar atrophy. Eventually, the patient developed gastrointestinal involvement. Using whole-exome sequencing (WES), we identified a novel homozygous frameshift mutation in the *TTC19* gene in the patient (NM_017775.3, c.581delG: p.Arg194Asnfs^*^16). Advanced genetic sequencing technologies developed in recent years have not only facilitated identification of novel disease genes, but also allowed revelations about novel phenotypes associated with mutations in the genes already linked with other clinical features. Our findings expanded the clinical features of *TTC19* mutation to potentially include gastrointestinal involvement. Further functional studies are needed to elucidate the underlying pathophysiological mechanisms.

## Background

Mitochondrial respiratory chain (MRC), consisting of five enzymatic complexes embedded in the inner mitochondrial membrane, has an important role in providing cells with ATP. Mutations in the nuclear or mitochondrial DNA affecting MRC could result in a wide array of disorders with various neurological and non-neurological presentations (1). Complex III, consisting of 11 subunits, is responsible for the transfer of electrons from reduced coenzyme Q to cytochrome C and thus, contributes to the formation of electrochemical potential ultimately leading to the production of ATP (2).

Mitochondrial complex III deficiency, nuclear type 2 (OMIM: 615157) is an autosomal-recessive disorder caused by mutations in *TTC19* gene. *TTC19* (Entrez Gene: 54902; OMIM: 613814) is involved in the preservation of complex III function by allowing turnover of Rieske protein through removal of the N-terminal proteolytic fragment of the protein (3). Mutations in *TTC19* have been shown to be linked with rapidly progressive neurological impairment (2), spinocerebellar ataxia (4–6), progressive psychosis (4), Leigh syndrome (7), developmental delay and regression in childhood (2, 8–10), bilateral cherry red spots, and failure to thrive (10). Clinical, neuroimaging, and biochemical findings in patients with pathogenic mutations in *TTC19* gene are summarized in Table 1.

**Table 1:** Clinical, neuroimaging, and biochemical findings in patients with *TTC19* mutations.

**References**	**Ghezzi** **et al**. **(****2****)**				**Nogueira** **et al**. **(****4****)**				**Atwal (7)**	**Morino** **et al. (5)**	**Melchionda** **et al. (8)**	**Kunii** **et al. (6)**	**Mordaunt** **et al. (10)**	**Koch** **et al**. **(****9****)**				**Conboy** **et al. (11)**	**The present study**
**Sex**	F	M	F	M	M	M	M	F	M	F	F	M	F	M	M	M	F	M	M
**Age at onset**	5 years	10 years	5 years	43 years	27 years	12 years	15 years	34 years	1 year	31 years	18 months	25 years	8 years	Neonatal	19 months	3 years	6 years	3.5 years	7 years
**Origin**	Italian	Italian	Italian	Italian	Portuguese	Portuguese	Portuguese	Portuguese	Hispanic	Japanese	Arab	Japanese	Iraqi	Turkish	Austrian	Romanian	Romanian	Kuwaiti	Iranian
**Presenting signs and symptoms**	Learning disability and gait ataxia	Learning disability and gait ataxia	Regression of language and gait ataxia	Weakness of all extremities	Mood disorder and gait ataxia	Compulsive lying	Aggressive behavior	Aggressive behavior	Developmental delay and language regression	Dysarthria	Unsteady gait with frequent falls	Mood disorder and gait ataxia	Developmental delay	Lactic acidosis	Global developmental delay, ataxia, dysarthria, hypotonia	Developmental delay, ataxia, regression, hypotonia	Mild developmental delay, hypotonia	Recurrent stroke-like episodes, developmental delay	Aggressive behavior and hyperactivity
**Elevated lactate (Blood/CSF)**	NA	NA	NA	NA	NA	NA	NA	NA	NA	+/NA	–/NA	–/–	–/–	+/NA	+/NA	–/–	NA	NA	+/NA
**Neurological findings**																			
Cognitive impairment	NA	+	NA	NA	+	+	+	+	NA	+	+	+	+	+	NA	NA	NA	+	+
Behavioral disorder	NA	NA	NA	NA	+	+	+	+	NA	NA	+	+	+	NA	NA	NA	–	NA	+
Ataxia	+	+	+	+	+	+	+	+	NA	+	+	+	+	+	+	+	–	+	+
Dysphagia	+	+	NA	NA	+	+	+	+	NA	NA	NA	NA	+	+	+	+	–	+	+
Dysarthria	+	+	+	+	+	+	+	+	NA	+	+	+	–	+	+	+	–	+	+
Spasticity	NA	NA	NA	+	+	+	+	NA	NA	NA	NA	+	–	+	+	+	–	NA	+
Epilepsy	NA	NA	NA	NA	NA	NA	NA	NA	NA	NA	NA	NA	–	+	+	+	–	NA	–
Hyperactive deep tendon reflexes	+	NA	NA	NA	+	+	+	+	NA	+	–	+	–	+	+	+	+	NA	+
**Neuroimaging features**	Leukoencephalopathy, hyper-intense caudate nucleus, and cerebellar atrophy	NA	Cerebellar atrophy	NA	Olivo-ponto-cerebellar atrophy and hypersignal changes in caudate, putamen, cerebellar dentate nucleus, medial midbrain, and medullary olives on T_2_-weighted sequences				Progressing T_2_ high signal lesions in putamen, caudate body, and brainstem	Cerebellar atrophy and bilateral T_2_ high intensity at inferior olives	Mild cerebellar vermis atrophy and bilateral symmetrical T_2_ high intensity lesions in lentiform nucleus with cavitated aspects on FLAIR sequence	Cerebellar atrophy and symmetric T_2_ high intensity lesions in the inferior olives and adjacent to periaqueductal gray matter	Mild cerebellar and cerebral volume loss, bilateral patchy high signal T_2_/FLAIR, and hypo- to iso-intense T_1_ foci within the lentiform nuclei	Symmetrical T_2_-weighted hyper-intensities of basal ganglia and periventricular white matter	Bilateral T_2_-weighted hyper-intensities of the putamen, the caudate nucleus, and the mesencephalon periaqueductal gray matter	Hyper-intensities of nucleus lenticularis and nucleus caudatus, loss of volume in the putamen, and cerebral atrophy	Hyper-intensities in caput caudate nucleus and basal parts of the putamen, increased interfoliar spaces in cerebellum	Bilateral symmetrical T_2_-weighted hyper-intensities and cystic changes of putamina and the caudate nuclei	Bilateral hypersignal changes in caudate bodies and lentiform nuclei on T2 and FLAIR, cerebellar atrophy

Herein, we report on a 15-year-old boy presenting with psychiatric symptoms, progressive ataxia, spastic paraparesis, bilious vomiting, and constipation with a novel homozygous frameshift mutation in *TTC19*.

## Case Presentation

The patient is a 15-year-old boy, who is the first and only child of consanguineous healthy parents who were first cousins. He initially presented at the age of 7 years with psychiatric symptoms including aggressive behavior and hyperactivity for which he was under treatment with methylphenidate (Ritalin), risperidone, olanzapine, and biperiden. The patient had normal psychomotor development until the age of 13 years, when he began to develop speech difficulty. Between the age of 13 and 14, he started to experience gait disturbance and difficulty walking, which progressed in the following year and made the patient wheelchair-bound. On physical examination, the patient was emaciated and cachectic; weighing 45 kg with a height of 165 cm, his body mass index (BMI) was 16.5 kg/m^2^. Neurological examination was significant for bilateral hyperactive deep tendon reflexes, severe ataxia, tremor, horizontal nystagmus, and spasticity, which was more pronounced in the lower extremities. The patient made limited eye contact and appeared to have intellectual impairment. In addition, musculoskeletal examination was notable for *pes cavus*.

**Figure 1:**
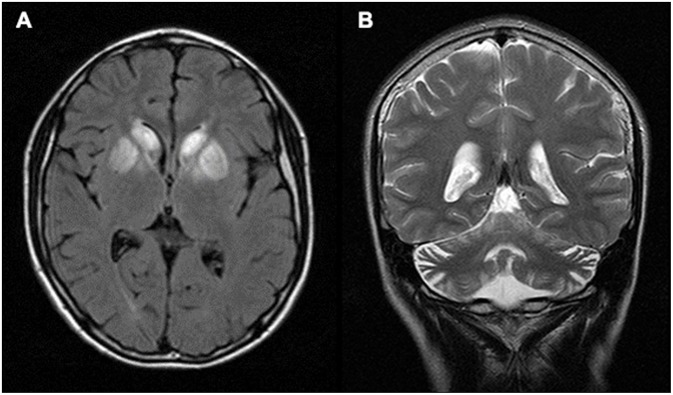
Brain MRI. **(A)** Axial FLAIR image of the patient showing hyper-signal changes bilaterally in caudate bodies and lentiform nuclei. **(B)** Coronal T_2_ image showing cerebellar atrophy.

Laboratory results including serum electrolytes, plasma ammonia, liver function test, blood amino-acid analysis, and cerebrospinal fluid examination were all normal. However, his blood lactate level was elevated to 27 mg/dL (reference range: 4.5–19.8 mg/dL). Magnetic resonance imaging (MRI) of the brain showed hypersignal changes bilaterally in caudate bodies and lentiform nuclei on T_2_ and fluid attenuated inversion recovery imaging (FLAIR). The lesions appeared as hypodensities in computed tomography (CT). In addition, cerebellar atrophy was detected (Figure 1).

The patient's condition deteriorated significantly in subsequent months, leading to severe cognitive impairment and mutism. In addition, the patient developed dysphagia, bilious vomiting, and constipation. Upper endoscopy revealed erythematous lesions distributed in the entire esophagus. Furthermore, gastric mucosa in the fundus, body, and antrum was hyperemic with multiple erosions. These lesions were also seen in the bulb and the second part of duodenum. A percutaneous endoscopic gastrostomy (PEG) tube was inserted due to feeding problems.

**Figure 2:**
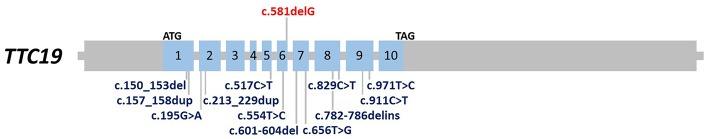
Schematic representation of *TTC19* gene (GenBank accession no. NM_017775.3). The blue boxes indicate exons. Novel (shown in red) and previously reported mutations are shown.

**Figure 3:**
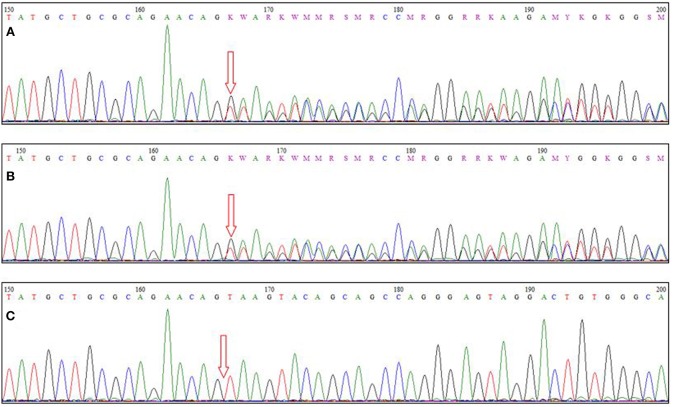
Sequence chromatograms of **(A,B)** parents and **(C)** the patient. The arrow indicates the site of the causative mutation.

Total genomic DNA was extracted from the patient's blood sample using QIAamp DNA Blood Mini kit (Qiagen, Germany). Subsequently, whole-exome sequencing (WES) was performed using Illumina NextSeq500 instrument. Variants with an allele frequency of more than 0.005 in gnomAD, EXAC, and our in-house database were excluded. In addition, synonymous and non-coding variants were filtered. Subsequently, considering the autosomal-recessive pattern of inheritance, homozygous, and compound heterozygous variants were analyzed (Table S1). Finally, correlation of the patient's clinical findings with phenotypes associated with the genes harboring identified genetic variations revealed that our patient was homozygous for a previously undescribed frameshift deletion mutation in *TTC19* gene (NM_017775.3: c.581delG: p.Arg194Asnfs^*^16). This novel mutation along with other previously reported pathogenic variants are represented in Figure 2. Sanger sequencing of exon six of the gene was also performed in the patient and his parents, using the following forward (5′-ATTCACAGTTGGCTCATCACTC-3′) and reverse (5′-AGATGTTGTGTGCCCCACTA-3′) primers. It was confirmed that both parents were heterozygous for the mutation, and that the proband was homozygous for this mutation (Figure 3).

Written informed consent was obtained from the patient's parents. This study was conducted in accordance with ethical standards of the declaration of Helsinki.

## Discussion and Conclusion

Mitochondrial disorders are clinically and genetically heterogeneous, greatly hindering the diagnosis of these disorders. Recent advances made in genetic technologies, allowing sequencing and in-depth investigation of the genome, has substantially improved the diagnosis of mitochondrial diseases. Autosomal-recessive cerebellar ataxias (ARCA) are a diverse group of neurodegenerative disorders characterized by movement incoordination and unsteadiness (12). A growing number of defects in biological pathways such as deficiency of DNA repair, defects in lipoprotein assembly, chaperone dysfunction, and mitochondrial defects can lead to ARCA (12). However, in almost half of the patients the genetic cause remains elusive (13). Herein, we reported a mitochondrial ARCA in a 15-year-old patient who presented with progressive ataxia, spastic paraparesis, psychiatric, and gastrointestinal symptoms with a novel frameshift deletion in *TTC19* gene. Protein-protein interaction analysis revealed an interaction of *TTC19* with many genes, including *ZFYVE26*, which has been identified as the cause of autosomal-recessive spastic paraplegia 15 (14, 15). Furthermore, animal models have highlighted the importance of *TTC19* gene. TTC19-null adult *Drosophilia melanogaster* exhibited reduced lifespan, low fertility, adult-onset motor impairment, and abnormal optomotor function (2).

The neurological and psychiatric manifestations of the patient described here coincides well with the manifestations reported earlier; progressive signs and symptoms of basal ganglia and cerebellar dysfunction such as ataxia, dysarthria, and spasticity were observed. Furthermore, psychiatric manifestations are often seen in dominant types of cerebellar ataxia, such as spinocerebellar ataxia 7 and 8; they are generally uncommon in those with ARCA (16, 17). However, it seems that patients with mutations in *TTC19* gene are more likely to present psychiatric manifestations, as described in our patient (4, 6).

Patients with mitochondrial disorders are frequently reported to have gastrointestinal symptoms. These symptoms might be the predominant presentation in disorders like mitochondrial neurogastrointestinal encephalomyopathy (MNGIE), whereas in other disorders like Leigh syndrome, gastrointestinal symptoms are usually less prominent compared with neurological presentations (18). Despite the fact that dysphagia is reported in previous reports, constipation and bilious vomiting have not hitherto been reported in the literature. This might be due to intestinal pseudo-obstruction, which has also been reported in other mitochondrial disorders (19, 20).

Advanced genetic sequencing technologies developed in recent years, such as WES, have not only facilitated identification of novel disease genes, but also allowed revelations about novel phenotypes associated with mutations in the genes already linked with other clinical features. Our findings expanded the clinical features of *TTC19* mutation to potentially include gastrointestinal involvement. Further functional studies are however needed to shed light over the underlying pathophysiological mechanisms.

## Data Availability

All data are available from the corresponding author on request.

## Ethics Statement

The Ethics Committee of the Persian BayanGene Research and Training Center approved the study protocol. The parents signed a written informed consent to participate in this study. Written informed consent was obtained from the parents of the patient for the publication of this case report.

## Author Contributions

MAF conceived and designed the study, collected, assembled, and interpreted NGS data. PH and SI clinically evaluated the patient. PH drafted the manuscript. SI, MAF, MY, and JB revised the manuscript. PH and JB did the bioinformatics analysis. MS, HD, MAFF, FS, ZF, and MR did the genetic studies.

### Conflict of Interest Statement

The authors declare that the research was conducted in the absence of any commercial or financial relationships that could be construed as a potential conflict of interest.
